# Strain-specific metabolic responses to long-term caloric restriction in female ILSXISS recombinant inbred mice

**DOI:** 10.1016/j.mce.2021.111376

**Published:** 2021-09-15

**Authors:** Lorna Mulvey, Stephen E. Wilkie, Gillian Borland, Kate Griffiths, Amy Sinclair, Dagmara McGuinness, David G. Watson, Colin Selman

**Affiliations:** a Institute of Biodiversity, Animal Health and Comparative Medicine, College of Medical, Veterinary and Life Sciences, University of Glasgow, Glasgow, G12 8QQ, UK; b Wellcome Centre for Integrative Parasitology, College of Medical, Veterinary and Life Sciences, Institute of Infection, Immunity and Inflammation, University of Glasgow, Glasgow, G12 8TA, UK; c Strathclyde Institute of Pharmacy and Biomedical Sciences, University of Strathclyde, The John Arbuthnott Building, 161 Cathedral Street, Glasgow, G4 0RE, UK

**Keywords:** Dietary restriction, White adipose tissue, Brown adipose tissue, Genetic heterogeneity, Metabolomics

## Abstract

The role that genetic background may play in the responsiveness of organisms to interventions such as caloric restriction (CR) is underappreciated but potentially important. We investigated the impact of genetic background on a suite of metabolic parameters in female recombinant inbred ILSXISS mouse strains previously reported to show divergent lifespan responses to 40% CR (TejJ89-lifespan extension; TejJ48-lifespan unaffected; TejJ114-lifespan shortening). Body mass was reduced across all strains following 10 months of 40% CR, although this loss (relative to *ad libitum* controls) was greater in TejJ114 relative to the other strains. Gonadal white adipose tissue (gWAT) mass was similarly reduced across all strains following 40% CR, but brown adipose tissue (BAT) mass increased only in strains TejJ89 and TejJ48. Surprisingly, glucose tolerance was improved most notably by CR in TejJ114, while both strains TejJ89 and TejJ114 were hyperinsulinemic following CR relative to their AL controls. We subsequently undertook an unbiased metabolomic approach in gWAT and BAT tissue derived from strains TejJ89 and TejJ114 mice under AL and 40% CR. In gWAT from TejJ89 a significant reduction in several long chain unsaturated fatty acids was observed following 40% CR, but gWAT from TejJ114 appeared relatively unresponsive to CR with far fewer metabolites changing. Phosphatidylethanoloamine lipids within the BAT were typically elevated in TejJ89 following CR, while some phosphatidylglycerol lipids were decreased. However, BAT from strain TejJ114 again appeared unresponsive to CR. These data highlight strain-specific metabolic differences exist in ILSXISS mice following 40% CR. We suggest that precisely how different fat depots respond dynamically to CR may be an important factor in the variable longevity under 40% CR reported in these mice.

## Highlights

• ILSXISS mice show strain-specific variation in longevity following CR.
• Enhanced glucose homeostasis did not correlate with reported longevity under CR.
• Shortened lifespan under CR was associated with relatively unresponsive WAT and BAT.
• How fat depots respond to CR may help explain lifespan differences in ILSXISS mice.

## 1 Introduction

It has been well established that restricting caloric intake (caloric restriction, CR), restricting specific dietary macro- or micro-nutrients, or restricting temporal food availability can extend lifespan and improve healthspan in a wide range of organisms (Fontana and Partridge, 2015; Speakman and Mitchell, 2011; Sinclair, 2005). Whilst the effects of CR appear highly conserved across taxa, the magnitude of the benefits observed appear to be affected by several factors, including sex, age at onset of CR, and both the duration and magnitude of the CR regime (Selman and Swindell, 2018; Swindell, 2012; Mitchell et al., 2016). In mice, much of what we currently understand about the physiological, cellular and molecular processes underlying CR has been revealed through studying male mice of the C57BL/6 strain (Selman and Swindell, 2018). However, what is becoming more and more apparent is that genetic background may underlie some of the phenotypic diversity observed following CR in mice (Selman and Swindell, 2018; Mitchell et al., 2016; Mulvey et al., 2014). Indeed, this may also be relevant to the CR response of both non-human primates and humans (Mattison et al., 2017).

Across different strains of mice, it is well established through employing comparative-type approaches that strain-specific differences in metabolic rate, body composition and glucose homeostasis occur under CR (Mitchell et al., 2016,Sohal et al., 2009,Mulvey et al., 2017,Ferguson et al., 2007,Forster et al., 2003,Hempenstall et al., 2010). In addition, several studies have reported that strain-specific effects exist in terms of the magnitude of lifespan extension achieved following CR in mice ((Selman and Swindell, 2018; Mulvey et al., 2014). For example, significant debate within the literature has existed regarding the lifespan response of DBA/2 mice to CR, with reported lifespan effects ranging from lifespan shortening, through to no effect, to lifespan extension relative to *ad libitum* (AL) controls (Forster et al., 2003; Fernandes et al., 1976; Bronson and Lipman, 1991; Turturro et al., 1999). In a recent large-scale study, it was shown that both male and female DBA/2J mice responded to CR, although the magnitude of lifespan effects under different CR regimes did not identically mirror the effects of CR seen in C57BL/6J mice (Mitchell et al., 2016). Further support for the importance of genetic background in the CR response was provided by studies in ILSXISS recombinant inbred mice (Liao et al., 2010; Rikke et al., 2010). In two separate studies undertaken by the Universities of Texas and Colorado, distinct lifespan differences were reported across different ILSXISS strains exposed to 40% CR, ranging from life-extension to life-shortening when compared to strain-specific AL controls. Despite limitations existing in each of these studies (Selman and Swindell, 2018), utilizing such strain-specificity responses may help identify candidate mechanisms underlying the beneficial effects of CR (Selman and Swindell, 2018; Swindell, 2012; Sohal et al., 2009; Mulvey et al., 2017). Indeed, the strain-specificity of these mice has been utilised previously to draw conclusions on how genotype may alter well characterised CR responses. Utilizing such an approach across different ILSXISS mice suggested that the maintenance of adiposity across strains exposed to CR was associated with lifespan extension under CR ((Liao et al., 2011), but see also (Speakman and Mitchell, 2011)). In addition, we have previously shown that female ILSXISS strains that differ in their longevity under 40% CR also show variation in their mitochondrial phenotypes under 40% CR (Mulvey et al., 2017).

Here we employed a comparative-type approach to further interrogate the strain-specific effects of long-term 40% CR (10 months duration) on a range of physiological parameters in female mice from three individual ILSXISS strains (strains TejJ89; TejJ48; TejJ114) under *ad libitum* (AL) feeding and 40% CR. We then utilised an unbiased approach to interrogate metabolite differences in both gonadal white adipose tissue and brown adipose tissue in strains TejJ89 and TejJ114. All three strains have previously been reported to show repeatable directional responses to 40% CR across two independent studies (Liao et al., 2010; Rikke et al., 2010) — from life-extension (TejJ89), through to no effect (TejJ48) through to life-shortening (TejJ114), relative to their strain-specific AL controls.

## 2 Materials and methods

### 2.1 Mice and caloric restriction protocol

Breeding pairs from three strains of ILSXISS mice (TejJ89; TejJ48; TejJ114) were purchased from a commercial producer (The Jackson Laboratory, Bar Harbor, Maine, URL:http://www.informatics.jax.org), then bred at The University of Glasgow to generate the experimental mice described here. As previously described in detail (Mulvey et al., 2017; Lee et al., 2019; Wilkie et al., 2020), female mice from these strains show repeatable effects of 40% on lifespan, with no differences in median lifespan reported across these strains under *ad libitum* (AL) conditions (Liao et al., 2010; Rikke et al., 2010). Female mice were maintained under identical conditions to those previously described (Mulvey et al., 2017), with all mice maintained from weaning onwards at 22 ± 2 °C in groups of 4 mice from the same strain within shoebox cages (48 cm × 15 cm × 13 cm). TejJ114 mice were significantly lighter than mice from strains TejJ89 (t = 4.569, p < 0.001) and TejJ48 (t = 6.539, p < 0.001) immediately prior the start of the experiment at 9 weeks of age. CR was introduced in a graded fashion-from 10% CR at 10 weeks of age to 40% CR from 12 weeks of age onwards. Each week, the total food intake of all AL mice from a particular strain was measured (±0.01 g) and then the food intake for the CR cohort of that strain calculated from the average AL intake per mouse over the pre-ceding week (Selman and Hempenstall, 2012). At 13 months of age (equivalent to 10 months of 40% CR) mice were culled by cervical dislocation. All mice (AL and CR) were fed a standard mouse chow throughout the experiment (CRM(P), Research Diets Services, LBS Biotech, UK; Atwater Fuel Energy-protein 22%, carbohydrate 69%, fat 9%). All procedures were undertaken under a licence from the UK Home Office (Project Licence 60/4504) and followed the “principles of laboratory animal care” (NIH Publication No. 86–23, revised 1985) and local ethical review (University of Glasgow). All studies described were undertaken in female mice.

### 2.2 Metabolic parameters

Body mass (±0.01 g) was recorded weekly between 10 and 12 weeks of age and then monthly thereafter. All mice were fasted overnight (1800hrs–0800hrs) following previously published protocols (Hempenstall et al., 2010; Selman et al., 2008) before glucose tolerance tests were undertaken. The next morning mice were placed in heat boxes for ~15 min to aid vasodilation and then a fasting blood glucose measurement (OneTouch Ultra, Lifespan, UK) was taken following tail venesection. Glucose tolerance was then recorded by measuring blood glucose following an IP injection of 20% glucose solution (2g of glucose/kg) at 15-, 30-, 60- and 120-mins post-injection. Glucose tolerance was expressed as the area under the curve over the 120 min period. Fed blood glucose was at 1100hrs and from CR mice at 1800hrs (following early feeding of CR mice at 1500hrs), to ensure mice were post-prandial (Hempenstall et al., 2010). Fasting plasma insulin levels were determined using a mouse insulin ELISA kit (EMD Millipore Corp, USA) and fasting plasma IGF-1 levels determined using a mouse IGF-1 ELISA (Quantikine R&D Systems Inc, USA). Insulin resistance was estimated using the updated homeostatic model assessment 2 (HOMA2) model (Wallace et al., 2004).

Brown adipose tissue and gonadal white adipose tissue were dissected immediately after death (13 months of age), weighed (±0.0001 g), frozen in liquid Nitrogen and subsequently stored at −80 °C until use.

### 2.3 Metabolomic analysis of WAT and BAT samples

An untargeted metabolomic analysis was undertaken in both gonadal white adipose tissue and brown adipose tissue derived from AL and 40% CR mice from strains TejJ89 and TejJ114. A total of 4 female mice were sampled for each treatment and tissue. Metabolomic analysis was not undertaken on either WAT or BAT from strain TejJ48. Analysis was undertaken using an Accela HPLC linked to an Exactive Orbitrap mass spectrometer (Thermo Fisher Scientific, Germany) and a hydrophilic interaction liquid chromatography (HILIC) column (ZIC-pHILIC, 150 × 4.6 mm, 5 μm particle size; Hichrom Ltd. UK), with all protocols described in full elsewhere (Alamri et al., 2018). Data collection employed the Xcalibur 2.1.0 software package (Thermo Fisher Scientific, UK), and MzMatch software (IDEOM) (Creek et al., 2012) was used to convert the signal peaks generated by Xcalibur into numeric values, with these values subsequently processed and analysed. Metabolites were detected to the metabolomics standards initiative (MSI) levels 1 or 2, as previously described (Alamri et al., 2018). All data were log-transformed and then analysed for significance using T tests in Excel, with SIMCA-P used for principal component analysis (PCA) comparisons. For all data, multiple comparison tests were run using the Benjamini Hochberg test. PCA plots were generated using MetaboAnalyst 5.0 (Chong et al., 2018).

### 2.4 Statistical analysis

Statistical analyses were performed using SPSS (SPSS Inc., USA, version 25) and GraphPad Prism (GraphPad Inc., USA, version 9) software. T-tests and general linear modelling (GLM) were used as appropriate. GLM used strain (TejJ89; TejJ48, TejJ114) and treatment (AL or CR) as fixed factors. In all cases, non-significant interaction effects (*p* > 0.05) in the GLM analyses were removed to obtain the best-fitted model. The Grubbs test was used to test for outliers (alpha = 0.05). While for some data the data presented are expressed as a percentage of total body mass (e.g. gonadal WAT and BAT), all statistical analyses were undertaken using raw data rather than using body mass corrected data with body mass introduced to the GLM as a co-variate. Post-hoc Tukey tests were used to examine significant strain-specific effects and t-tests used to examine treatment effects within a strain. Results are reported as mean ± standard error of the mean (SEM), with *p* < 0.05 regarded as statistically significant. Treatment effects are denoted by *p < 0.05; **p < 0.01; ***p < 0.001 and strain effects are denoted by ^s^*p* < 0.05;^ss^*p*<0.01;^sss^*p*<0.001.

## 3 Results

**Fig. 1:**
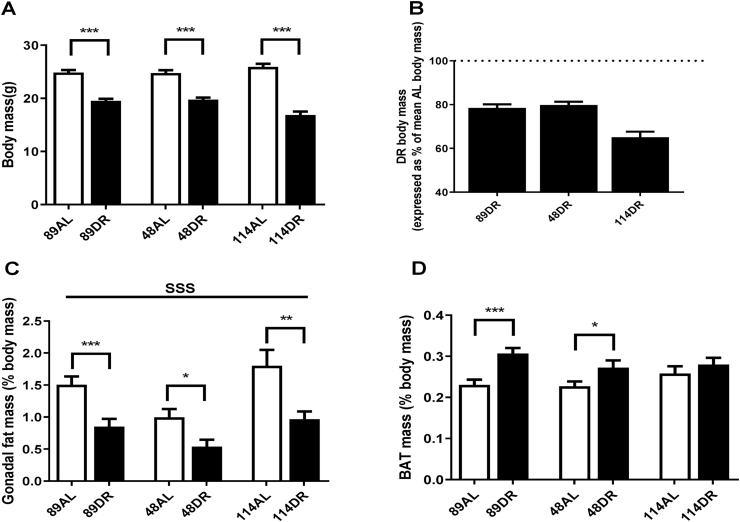
Total body mass (g) (A, where n = 11–20 per group), reduction in body mass in caloric restriction (CR) mice relative to *Ad libitum* (AL) mice (expressed as a percentage of mean strain-specific AL body mass (B, where n = 11–20 per group), Gonadal fat mass (C, expressed as a percentage of body mass, where n = 10–17 per group) and Brown adipose tissue (BAT) mass (D, expressed as a percentage of body mass, where n = 9–17 per group) in female TejJ89, TejJ48 and TejJ114 mice under *Ad libitum* and 40% caloric restriction (CR). Values are mean ± standard error of the mean (SEM). Treatment effects are denoted by *p < 0.05; **p < 0.01; ***p < 0.001 and strain effects are denoted by ^sss^*p*<0.001.

All ILSXISS strains showed a highly significant decrease in body mass following 10 months of 40% CR relative to their respective AL controls (Fig. 1A and S1A-C). When comparing across strains, a significant effect of treatment (F = 210.567, p < 0.001) on body mass was observed but no strain-specific effect was seen (F = 1.585, p = 0.211), although a significant treatment*strain interaction was detected (F = 8.640, p < 0.001). On further investigation, body mass did not differ between strains on AL feeding (F = 1.293, p = 0.285) but did significantly differ between strains under 40% CR (F = 9.708, p < 0.001, with TejJ114 mice being significantly lighter than both TejJ89 (p < 0.01) and TejJ48 (p < 0.001) mice. The loss in body mass following 10 months of 40% CR (expressed as % of AL body mass) in strains TejJ89 and TejJ48 was relatively similar (Fig. 1B) with these mice being able to maintain their BM around 80% of that of their AL controls. However, the loss in body mass of strain TejJ114 under CR was much more pronounced (Fig. 1B), with body mass being only around 65% of AL controls following 10 months of a 40% CR diet. Consequently, TejJ114 fell in the lower extremes of body mass maintenance under 40% CR as reported elsewhere (Rikke et al., 2006). However, the mass of gonadal white adipose tissue (gWAT) was similarly reduced by 40% CR across all 3 strains (Fig. 1C). A significant treatment (F = 25.612, p < 0.001) and strain (F = 7.195, P = 0.001) effect was detected, with strain TejJ48 having relatively smaller gWAT mass under both AL and CR conditions relative to TejJ89 and TejJ114. BAT mass (Fig. 1D) was increased under 40% CR (F = 15.337, p < 0.001) but no strain-specific differences were observed (F = 1.049, p = 0.356), although when further investigated using pair-wise comparisons the CR-induced BAT hypertrophy was significant in strains TejJ89 (p < 0.001) and TejJ48 (p = 0.040), but not in TejJ114 (p = 0.394).

**Fig. 2:**
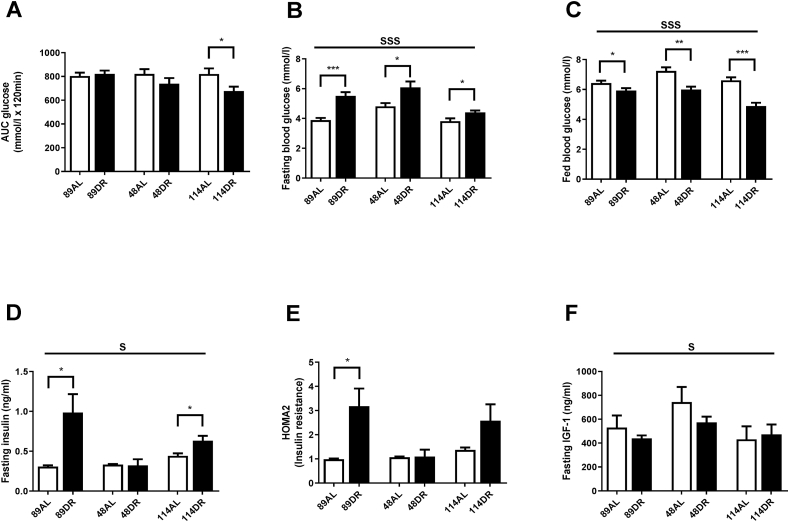
Glucose tolerance, fasting plasma insulin, insulin resistance and fasting plasma IGF-1 levels in female TejJ89, TejJ48 and TejJ114 mice under *Ad libitum* and 40% caloric restriction (CR). (A) Glucose tolerance (denoted by area under the curve (AUC) following a glucose injection, where n = 11–15 per group), (B) Fasting blood glucose (where n = 11–20 per group, (C) Fed blood glucose (where n = 11–12 per group), (D) Fasting plasma insulin levels (where n = 5–7 per group), (E) Insulin resistance as determined by HOMA2 (where n = 5–7 per group), (F) Fasting plasma IGF-1 levels (where n = 5–7 per group). Values are mean ± standard error of the mean (SEM). Treatment effects are denoted by *p < 0.05; **p < 0.01; ***p < 0.001 and strain effects are denoted by ^s^*p* < 0.05; ^sss^*p*<0.001.

Comparing across strains, an overall treatment (F = 5.262, p = 0.025), but no genotype effect (F = 1.209, p = 0.305), was observed on glucose tolerance. Using pairwise comparisons a significantly improved glucose tolerance following 40% CR was observed only in strain TejJ114 (Fig. 2A and S2A-C). Fasting blood glucose levels (Fig. 2B) were elevated by 40% CR in all strains (F = 34.458, p < 0.001), with a significant strain effect also detected (F = 9.852, p < 0.001). Fed blood glucose levels (Fig. 2C) were significantly reduced under 40% CR in all 3 strains (F = 49.854, p < 0.001), with both a strain (F = 9.364, p < 0.001) and treatment*strain interaction (F = 4.887, p = 0.011) effect detected. Significant treatment (F = 8.463, p = 0.007), strain (F = 3.814, p = 0.033) and treatment*strain interaction (F = 4.440, p = 0.020) effects were observed on fasting plasma insulin levels (Fig. 2D). Surprisingly, both TejJ89 (p = 0.034) and TejJ114 (p = 0.033) were relatively hyperinsulinemic under 40% CR relative to their respective AL controls, although no treatment effect was detected in strain TejJ48 (p = 0.896). This was reflected by a significant treatment effect on HOMA2 insulin resistance (IR) (Fig. 2E) (F = 7.510, p = 0.010), although a pair-wise comparison only detected a significant effect on HOMA2 IR between AL and CR mice from strain TejJ89 (p = 0.031). The effect of strain on HOMA2 was not significant (F = 2.675, p = 0.084). Fasting plasma IGF-1 levels (Fig. 2F) were unaffected by CR (F = 1.344, p = 0.255), but a strain-specific difference in IGF-1 levels was detected (F = 3.354, p = 0.047), being relatively higher in strain TejJ48.

**Fig. 3:**
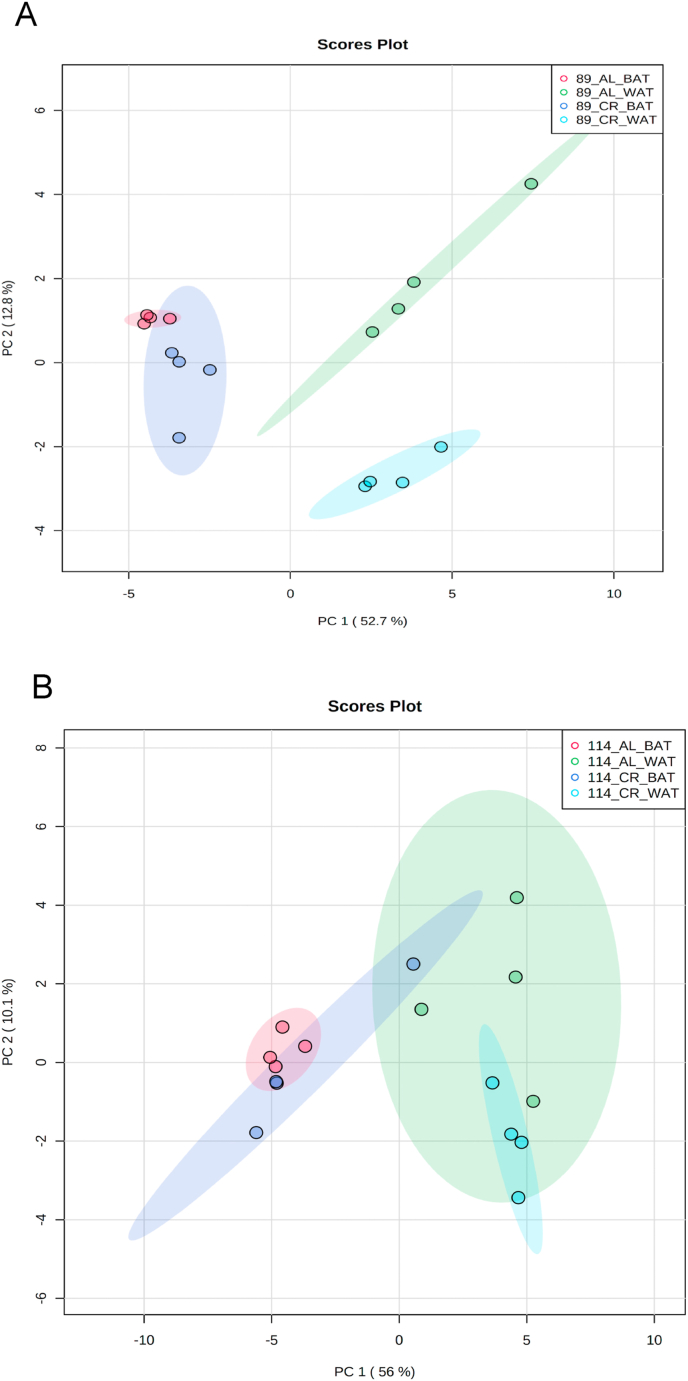
Scores plot between the selected PCs. Scores are defined as weighted average of the original variables in white adipose tissue (WAT) and brown adipose tissue (BAT) derived from *ad libitum* (AL) and 40% caloric restricted (CR) mice from strains TejJ89 (A) and TejJ114 (B). n = 4 female mice per group.

Given the strain-specific differences we observed in gWAT and the lack of CR-induced BAT hypertrophy in TejJ114 we undertook an unbiased metabolomic approach in gWAT and BAT tissue derived from strains TejJ89 and TejJ114 female mice under both AL and 40% CR, but not TejJ89. In Table 1 (see also Fig. 3A), the metabolomic data has been ordered according to the most significant changes in gWAT when comparing between AL TejJ89 and CR TejJ89 mice. In TejJ89 mice, a large proportion of the metabolites that were significantly altered under CR in gWAT were reduced (41/51 in total). Many of these were long chain polyunsaturated fatty acids (Table 1) including linoleic acid, docosahexaenoic acid suggesting enhanced fatty acid B-oxidation under CR in this strain. In addition, several amino acids associated with the Krebs cycle were also reduced in gWAT of TejJ89 under CR (e.g. L-Alanine, L-Valine, L-Glutamate, L-Leucine), alongside metabolites associated with Aminoacyl-tRNA biosynthesis, Pantothenate and CoA biosynthesis, and D-Glutamine and D-Glutamate metabolism. In contrast, far fewer metabolites (total of 30) were significantly altered in gWAT of strain TejJ114 under CR (Table 2), with 17 of these metabolites also changed in the same direction in TejJ89 WAT. However, the impact of CR on long chain fatty acid levels in gWAT from TejJ114 were again much less marked than in TejJ89 mice, although several phosphatidylethanolamine lipids were elevated under CR (Table 1, Table 2; Fig. 3B) across both strains. There was no evidence that the same amino acids affected in TejJ89 under CR were affected by CR in TejJ114. Indeed, there appeared to be very little distinction in the metabolite profiles in the WAT from strain TejJ114 under AL and CR feeding (Fig. 3B). In contrast to TejJ89, most of the metabolites affected by CR in gWAT from TejJ114 were increased (20/30 in total). We then compared the metabolite changes in BAT derived from TejJ89 mice (Table 3, Fig. 3A), finding a total of 49 metabolites affected by CR, with 30/49 significantly decreased in CR mice. Of these, several phospholipids, most notably phosphatidylglycerol (PG) lipids were decreased under CR, with PGs acting as important precursors in the synthesis of cardiolipin. The branch-chain amino acids L-leucine and L-valine were also significantly reduced under CR in the BAT of TejJ89 mice. In contrast several phosphatidylethanolamine (PE) lipids were elevated under CR in TeJ89 BAT, as was pantothenic acid which may indicate increased biosynthesis of CoA and lipolysis. As we previously reported, we observed no evidence of BAT hypertrophy under CR in TejJ114, and TejJ114 mice did not show the same changes within their BAT following CR (Fig. 3B). Indeed only 2 metabolites (PE32:1, LPE18:1 ether) were significantly affected by 30% CR (Table 3; Fig. 3B), and the direction of change of LPE18:1 (UP) was opposite to that seen in TejJ89 BAT (DOWN). In agreement, there appeared to be little distinction in the metabolite profiles in BAT under AL and CR conditions in strain TejJ114 (Fig. 3B).

**Table 1:**
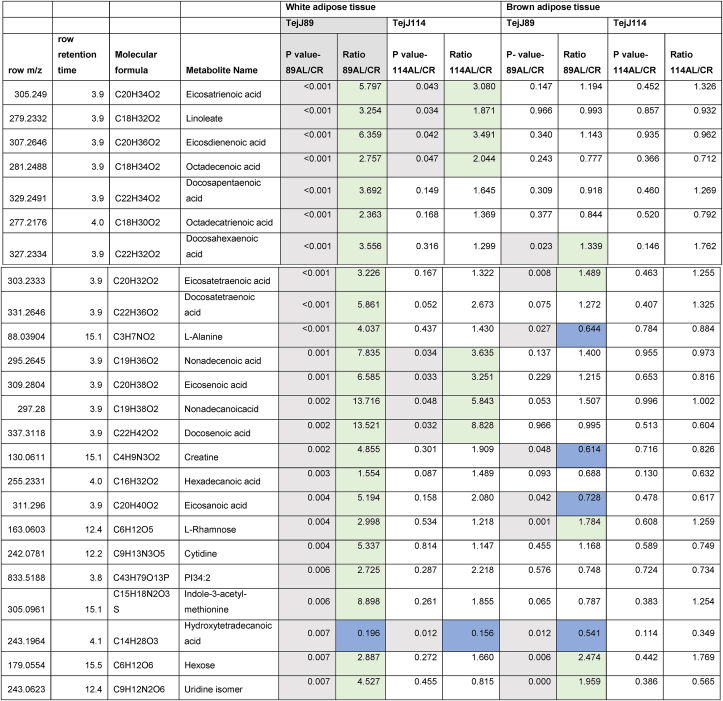

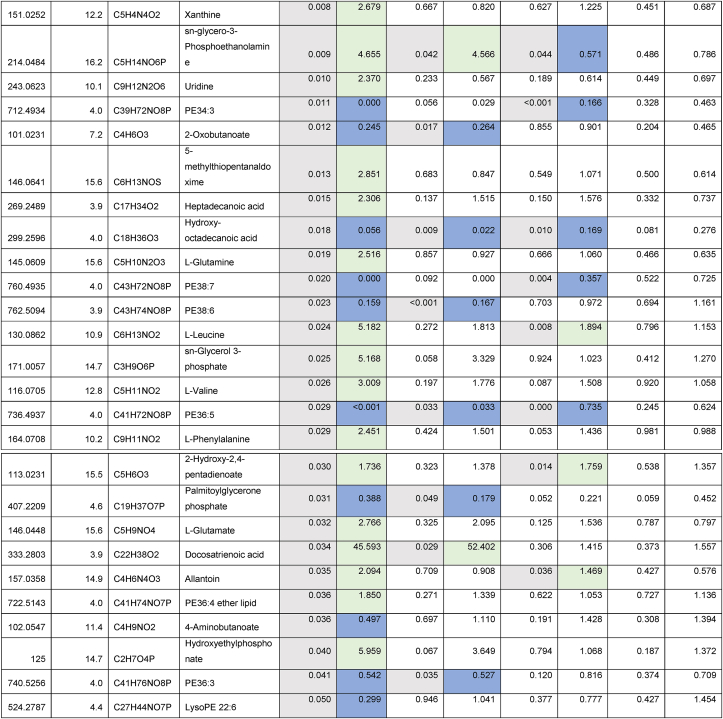
Metabolomic (Negative ion) data from white adipose tissue (WAT) and brown adipose tissue (BAT) derived from *ad libitum* (AL) and 40% caloric restricted (CR) mice from strains TejJ89 and TejJ114. Data ordered according to P values for TejJ89 gWAT AL vs CR. Grey box denotes significant (p < 0.05) changes, green box denotes DECREASE in metabolite in CR relative to AL mice and blue box denotes INCREASE in metabolite in CR relative to AL mice.

**Table 2:**
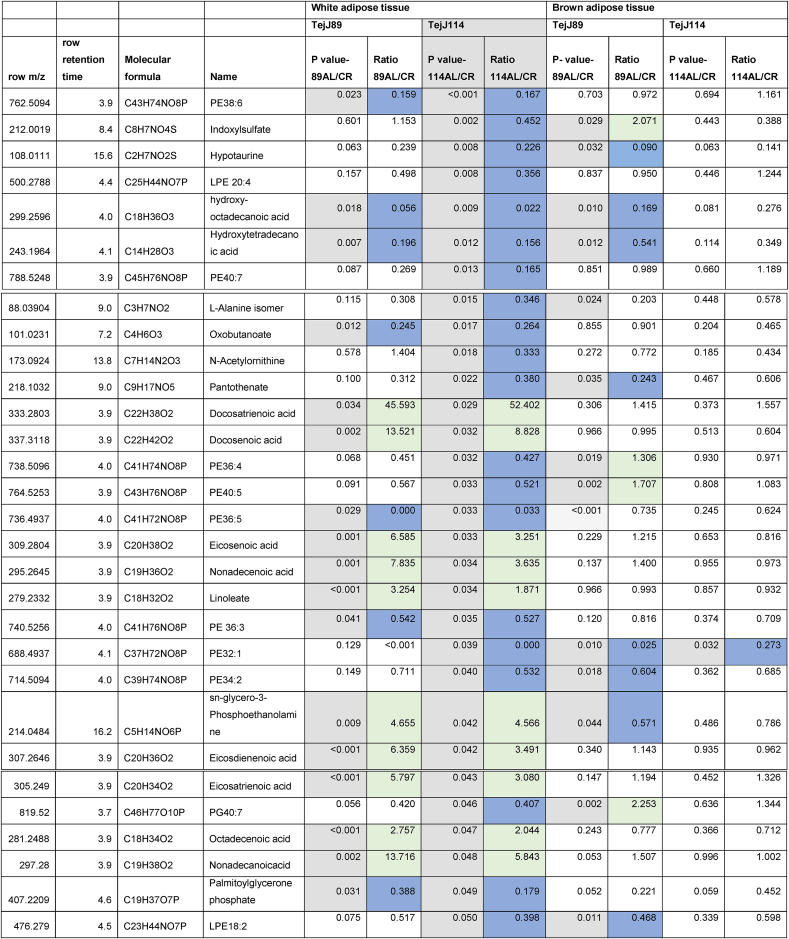
Metabolomic (Negative ion) data from white adipose tissue (WAT) and brown adipose tissue (BAT) derived from *ad libitum* (AL) and 40% caloric restricted (CR) mice from strains TejJ89 and TejJ114. Data ordered according to P values for TejJ114 gWAT AL vs CR. Grey box denotes significant (p < 0.05) changes, green box denotes DECREASE in metabolite in CR relative to AL mice and blue box denotes INCREASE in metabolite in CR relative to AL mice.

**Table 3:**
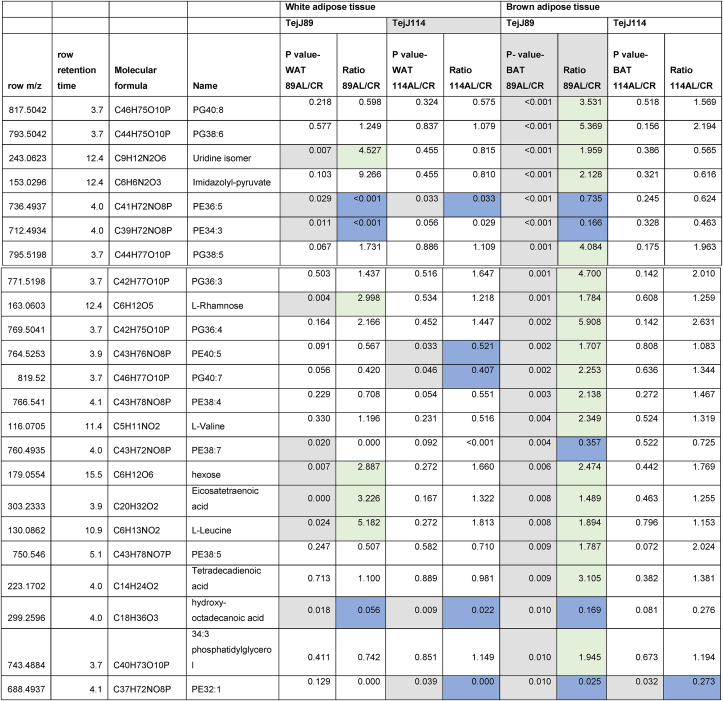

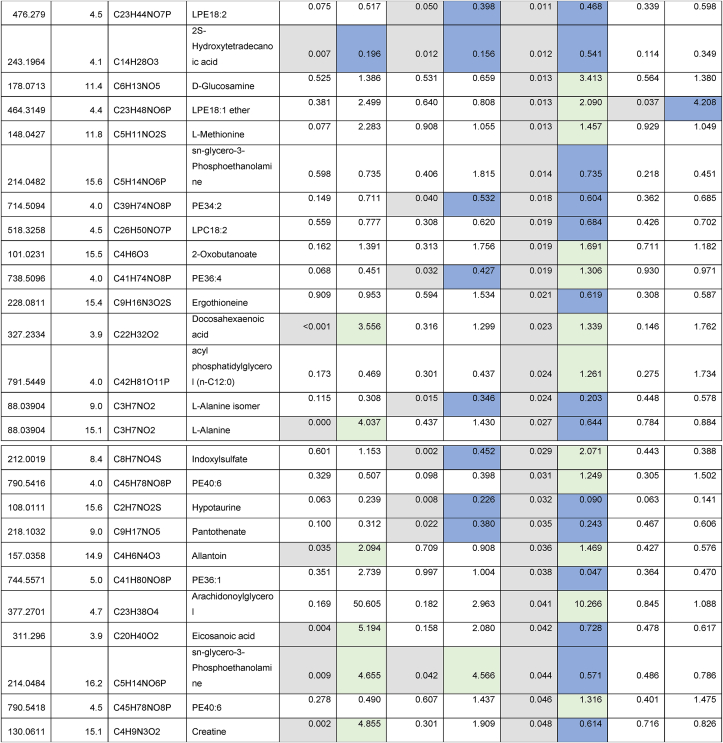
Metabolomic (Negative ion) data from white adipose tissue (WAT) and brown adipose tissue (BAT) derived from *ad libitum* (AL) and 40% caloric restricted (CR) mice from strains TejJ89 and TejJ114. Data ordered according to p Values for TejJ89 BAT AL vs CR. Grey box denotes significant (p < 0.05) changes, green box denotes DECREASE in metabolite in CR relative to AL mice and blue box denotes INCREASE in metabolite in CR relative to AL mice.

## 4 Discussion

While the beneficial impact of caloric restriction on both healthspan and lifespan in mice is well established, much of what we know has typically been described through studies employing a very small number of genetic strains and in a single sex, the male (Selman and Swindell, 2018). Consequently, there is a real need to expand the choice of mouse strains used in ageing studies, including caloric restriction (CR). Investigations of how interventions that modulate ageing impact on females in addition to males are also urgently required. It has previously been reported that genetic background plays an important role in the metabolic response to CR in mice (Mitchell et al., 2016; Hempenstall et al., 2010; Gelegen et al., 2006), and that longevity under CR is not affected to the same extent in different mouse strains (Mitchell et al., 2016; Forster et al., 2003; Liao et al., 2010; Rikke et al., 2010). Even within the same mouse strain the beneficial effects of CR can vary in a sex-specific manner (Mitchell et al., 2016). Consequently, we examined a suite of metabolic parameters in different strains of female recombinant inbred ILSXISS mice which have previously been reported to show strain-specific variation in longevity under 40% CR (Liao et al., 2010; Rikke et al., 2010).

### 4.1 The impact of CR on white adipose tissue (WAT)

It has been reported in rats that those individuals that lose the least amount of fat under CR live the longest (Bertrand et al., 1980), although the universality of this relationship has been brought in to question (for full discussion see (Speakman and Mitchell, 2011; Swindell, 2012)). In ILSXISS strains, it has previously been reported that those strains that showed the greatest lifespan extension under 40% CR also lost the least amount of fat relative to their strain-specific AL controls when measured at 15–17 months and 20–22 months of age (Liao et al., 2011). In other words, the ability to maintain adiposity under CR may be important to CR-induced longevity. We observed a significant decline in body mass following 40% CR in all three ILSXISS strains (TejJ89, TejJ48 and TejJ114) relative to their strain-specific AL controls. However, the loss in BM was significantly greater in TejJ114 relative to its AL controls compared to the loss in BM observed in the other 2 strains under CR. gWAT also similarly decreased in mass across all 3 strains under CR, although strain TejJ48 had a significantly reduced gWAT mass under both AL and CR feeding compared to the other 2 strains. TejJ114, which has previously been reported to show a shortening in lifespan under 40% CR (Liao et al., 2010; Rikke et al., 2010) did not lose proportionally more gWAT compared to strains TejJ89 and TejJ48 under 40% CR, although they did lose proportionally more body mass. Of course, we did not measure total fat mass in this study, but gWAT has been shown to be a highly accurate predictor of whole body adiposity (Oldknow et al., 2015). Specific WAT stores may respond differently to CR and it has previously been reported that gonadal (epididymal) fat in male C57BL/6J mice was preferentially utilised during short-term CR, to a much greater degree that other fat stores (Mitchell et al., 2015a). Female mice may also preferentially maintain their subcutaneous fat stores under CR, unlike male mice (Shi et al., 2007). It should also be noted that CR also significantly impacts total lean mass and individual organ mass (Hempenstall et al., 2010; Mitchell et al., 2015a; Selman et al., 2005; Even et al., 2001), and so while TejJ114 lost proportionally more mass under 40% CR we cannot be certain at this time where this loss occurred.

### 4.2 The metabolomic signature within WAT following CR

We subsequently utilised an unbiased metabolomic assessment of gWAT derived from TejJ89 and TejJ114 under AL and CR feeding to determine whether the metabolic signature of this tissue differed under CR across these strains. The metabolic profiles affected by CR in strains TejJ89 and TejJ114 when compared to gWAT from their strain-specific AL controls were quite distinct, with gWAT from TejJ114 being far less responsive to CR than strain TejJ89. It appears that TejJ89 may rely to a far greater extent on fatty acid catabolism and β-oxidation during CR, with many metabolites associated with biosynthesis of unsaturated fatty acids reduced. This may then indicate a greater flux in free fatty acids from adipose tissue in TejJ89 under CR to help fuel the TCA cycle within the liver at this time, as suggested elsewhere as being potentially critical to eliciting the lifespan and healthspan benefits of CR (Mitchell et al., 2016). This metabolic switch under CR appears to be lacking in gWAT from TejJ114, and we have previously reported that TejJ114 mice show evidence of hepatic mitochondrial dysfunction under 40% CR (Mulvey et al., 2017). Interestingly, the metabolite indoxyl sulfate was elevated in gWAT from TejJ114 mice under CR. Indoxyl sulfate is a metabolite of L-tryptophan and is an established uremic- and cardio-toxin that has been linked to adipose tissue inflammation and oxidative stress (Tanaka et al., 2020). Our principal component analysis (PCA) indicated that there is significant overlap in the metabolic profiles between TejJ89 and TejJ114 under AL feeding, and that the separation between strains becomes much more apparent following CR. However, several phosphatidylethanolamine (PE) lipids were elevated in common across both strains under CR. These lipids account for ~25% of phospholipids in mammals and have many cellular functions, including roles in autophagy, oxidative phosphorylation, antioxidant protection and mitochondrial stability (Vance and Tasseva, 2013; Calzada et al., 2016).

### 4.3 The impact of CR on brown adipose tissue (BAT)

Body temperature typically reduces in mammals subject to CR (Weindruch and Walford, 1988). It is thought that non-shivering thermogenesis (NST) within the brown adipose tissue (BAT) may be of particular importance in maintaining energy balance, particularly in animals like mice that employ periodic torpor during CR to help achieve this (Green et al., 2020). It has also been suggested that BAT activation may be required during CR in mice to increase body temperature during periods of intense and acute activity, such as seen in anticipation of feeding (Green et al., 2020). We observed CR-induced hypertrophy in BAT mass under 40% CR but significantly so only in strains TejJ89 and TejJ48, and not in TejJ114. We have previously reported BAT hypertrophy in male Fischer rats under lifelong 40% CR (Selman et al., 2005), but other studies in mice reported no effect (male ICR mice (Elsukova et al., 2012)) or a reduction in BAT mass under CR (male C57BL/6J mice (Mitchell et al., 2015a), male 129S2/SvPasCrl mice (Corrales et al., 2019). However, in the latter study CR did appear to preserve aspects of BAT function during ageing and promoted browning within subcutaneous WAT (Corrales et al., 2019). Surprisingly few studies have examined how exactly BAT mass and function is affected by CR in female mice (see (Hoffman and Valencak, 2021)). Consequently, it is currently unclear whether the hypertrophy we see in TejJ89 and TejJ48 is typical of female mice under CR or is simply specific to these ILSXISS strains. Interestingly, long-lived dwarf GHRKO (Li et al., 2003) and Ames (Darcy et al., 2016) mice possess proportionally larger and more functionally active BAT depots relative to control mice and this has been suggested as being an important factor in their exceptional longevity (Darcy and Bartke, 2017; Valencak et al., 2020). We also found that the n-3 PUFA docosahexaenoic acid (DHA) was reduced significantly in both WAT and BAT of TejJ89 mice under CR but unaltered in both tissues under CR in TejJ114 mice. High levels of n-3 PUFAs are associated with oxidative stress, with several studies showing that low levels of DHA correlate positively with lifespan in mammals (Valencak et al., 2020; Valencak and Ruf, 2007; Hulbert et al., 2006; Hulbert et al., 2006; Hulbert et al., 2007). DHA is also reduced in multiple tissues following short-term CR in male Quackenbush Swiss mice (Faulks et al., 2006). We have previously shown that hepatic oxidative damage was reduced by CR in TejJ89 mice but increased by CR in TejJ114 mice (Mulvey et al., 2017), although we do not currently know if n-3 PUFAs were altered by CR within the liver. It has previously been reported that core body temperature in aged C57BL/6J mice correlated positively with lifespan (Reynolds et al., 1985). Significant variation in body temperature exists following CR in mice (Rikke et al., 2003), and intriguingly female ILSXISS strains that showed the most pronounced reductions in body temperature under 40% CR were also more likely to exhibit a shortening of lifespan under 40% CR (Liao et al., 2011). It is feasible that strain TejJ114 cannot maintain homeothermy under CR as efficiently as the other strains under CR. Crosstalk exists between different fat depots and it has been shown that surgical removal of BAT in Ames dwarf mice leads to a reduction in WAT depots, possibly because the WAT is required to fuel homeothermy (Darcy et al., 2016). Consequently, the absence of BAT hypertrophy in TejJ114 under CR may directly affect other fat depots, although this requires further investigation.

### 4.4 The metabolomic signature within BAT following CR

We also investigated the BAT metabolome under AL and CR feeding in strains TejJ89 and TejJ114. It has previously been reported that metabolomic analysis in male C57BL/6J mice identified several metabolites altered by CR, including a number associated with the TCA cycle, antioxidant protection and fatty acid degradation, several of which correlated with both body temperature and food anticipatory activity (Green et al., 2020). In TejJ89, several PE lipids were elevated within the BAT under CR, as seen with WAT under CR in both strains. Mitochondria have the capacity to synthesise PE lipids via the Mitochondrial Phosphatidylserine Decarboxylase Pathway (Calzada et al., 2016) and the inner mitochondrial membrane is highly enriched with PE lipids (Vance and Tasseva, 2013). In addition, dietary supplementation with plasmalogens, that include PE lipids, increased mitochondrial copy number, enhanced mitochondrial function and thermogenesis in BAT from mice maintained in the cold (Park et al., 2019). Several phosphatidylglycerol (PG) metabolites were reduced in the BAT in TejJ89 mice following CR. PGs are involved in cardiolipin synthesis and consequently play a key role in mitochondrial membrane biosynthesis, with an increase in BAT PGs, rather than a decrease as seen in our study, being associated with thermogenesis following cold exposure in mice (Lynes et al., 2018). In addition, the branch-chain amino acids (BCAAs) Leucine and Valine were reduced in the BAT from TejJ89 mice. BCAAs have been associated with a range of deleterious metabolic health effects (Yu et al., 2021), can act as substrates for non-shivering thermogenesis in BAT (Yoneshiro et al., 2019), and using enrichment analysis it was previously shown that BCAA degradative pathways were enhanced in WAT and BAT from long-lived Ames dwarf mice (Darcy et al., 2020). In contrast to previously published data (Green et al., 2020), there was little evidence that metabolites with antioxidant properties were elevated by CR in our ILSXISS strains. However, hypotaurine which does have reported antioxidant properties (Aruoma et al., 1988) and can elicit beneficial effects on mitochondrial function in model organisms (Wan et al., 2020) was elevated under CR in both WAT of TejJ114 and BAT of TejJ89 mice. In agreement with our findings in gWAT, the BAT metabolomic signature in TejJ114 was essentially unresponsive to 40% CR.

### 4.5 CR and glucose homeostasis in female ILSXISS mice

It is well established that CR induces many beneficial effects on glucose homeostasis, including improvements in glucose tolerance, a lowering of fed and fasting blood glucose levels and increased insulin sensitivity (Mitchell et al., 2016; Hempenstall et al., 2010; Selman and Hempenstall, 2012; Weindruch and Walford, 1988; Velingkaar et al., 2020). Overall, CR did improve glucose tolerance and reduce fed blood glucose levels across strains, in agreement with previous studies. However, the improvement in glucose tolerance under CR was most notable in TejJ114 (and not TejJ89), with fasting blood glucose levels increased by CR across all strains. In addition, while strain-specific differences were observed in both fasting insulin and fasting IGF-1 levels, the effects of CR were much less apparent. Fasting insulin levels increased under 40% CR in both TejJ89 and TejJ114, and TejJ89 mice were also more insulin resistant under 40% CR. While enhanced glucose tolerance, reduced fasting insulin and IGF-1 levels, and insulin sensitivity is a commonly described phenotype of long-lived mouse models, this is not always the case (Selman et al., 2008; Mitchell et al., 2015b; Dommel et al., 2021; Yu et al., 2019) and infers that improved glucose homeostasis may not be a prerequisite for longevity in mice. However, it should be noted that the strain-specific responses identified here are perhaps not surprising considering highly variable strain-specific metabolic phenotypes previously described for female ILSXISS mice following high fat diet feeding (Stockli et al., 2017). The effects of CR on glucose homeostasis are far less studied in female mice, with the improvements in glucose homeostasis and reductions in fasting insulin levels seen in C57BL/6J females on 20% CR, relative to AL controls, not further affected by 40% CR (Mitchell et al., 2016). Interestingly, glucose tolerance, fasting glucose and insulin levels in female C57BL/6J mice following 40% CR appeared much less responsive to dietary switches compared to male mice (Cameron et al., 2012).

## 5 Conclusions

Despite the lifespan benefits of CR being first demonstrated over 100 years ago (Osborne et al., 1917), precisely how CR acts mechanistically to elicit its beneficial effects on both lifespan and healthspan are still unclear. What has also become apparent over the past couple of decades is that the extent of CR-induced improvements on the individual depends on a number of factors. These factors include sex, duration of CR protocol, nature of CR (or dietary restriction) protocol, age of onset of CR and genetic background (Speakman and Mitchell, 2011; Selman and Swindell, 2018; Mulvey et al., 2014). To this end, recombinant inbred ILSXISS mice that show variable lifespan responses are a useful comparative tool, to try and disentangle what mechanisms may underlie CR-induced longevity (Mulvey et al., 2014, 2017; Liao et al., 2010, 2011; Rikke et al., 2003, 2010; Rikke and Johnson, 2007). Surprisingly, we saw relatively little benefit of long-term 40% CR on a range of metabolic parameters in female mice, and in those parameters such as glucose tolerance and insulin resistance that did change with CR, typically did not correlate as predicted with the reported CR-induced longevity. That is that improved glucose tolerance, reduced plasma insulin and reduced insulin resistance were not seen in the reported CR responding strain TejJ89. We did however find that CR had a significant effect on both WAT and BAT metabolite profiles in strain TejJ89 but that the effect of CR on these tissues in strain TejJ114, which reportedly shows a reduction of lifespan (relative to AL control) under 40% CR was much less responsive to CR. Consequently, it may be that *qualitative* differences in fat stores, their ultimate function-be that as a labile energy store or for non-shivering thermogenesis-may help explain strain-specificity in ILSXISS mice to 40% CR. Of course, our findings only examined a small number of strains and only studied one level of CR but do suggest that how particular fat depots respond to CR may be important in CR-induced longevity (Mitchell et al., 2015a, Mitchell et al., 2015b; Shi et al., 2007). What is clear is that in order to distinguish private and public mechanisms of ageing (Partridge and Gems, 2002), there is a need to increase the number of studies that examine CR using diverse genetic backgrounds in rodents (Selman and Swindell, 2018). However, in order to better understand how CR impact on lifespan and healthspan, there also needs to be more studies using female mice (Hoffman and Valencak, 2021). This will help determine whether the findings we see here on glucose homeostasis and on metabolite signatures are a general response to CR in female mice or simply something distinct to ILSXISS strains.
